# Application of Digital Games for Speech Therapy in Children: A Systematic Review of Features and Challenges

**DOI:** 10.1155/2022/4814945

**Published:** 2022-04-25

**Authors:** Soheila Saeedi, Hamid Bouraghi, Mohammad-Sadegh Seifpanahi, Marjan Ghazisaeedi

**Affiliations:** ^1^Department of Health Information Management and Medical Informatics, School of Allied Medical Sciences, Tehran University of Medical Sciences, Tehran, Iran; ^2^Department of Health Information Technology, School of Allied Medical Sciences, Hamadan University of Medical Sciences, Hamadan, Iran; ^3^Department of Speech and Language Pathology, Autism Spectrum Disorders Research Center, Hamadan University of Medical Sciences, Hamadan, Iran

## Abstract

**Introduction:**

Treatment of speech disorders during childhood is essential. Many technologies can help speech and language pathologists (SLPs) to practice speech skills, one of which is digital games. This study aimed to systematically investigate the games developed to treat speech disorders and their challenges in children.

**Methods:**

A comprehensive search was conducted in four databases, including Medline (through PubMed), Scopus, Web of Science, and IEEE Xplore, to retrieve English articles published by July 14, 2021. The articles in which a digital game was developed to treat speech disorders in children were included in the study. Then, the features of the designed games and their challenges were extracted from the studies.

**Results:**

After reviewing the full texts of 69 articles and assessing them in terms of inclusion and exclusion criteria, 27 articles were included in the systematic review. In these articles, 59.25% of the games had been developed in English language and children with hearing impairments had received much attention from researchers compared to other patients. Also, the Mel-Frequency Cepstral Coefficients (MFCC) algorithm and the PocketSphinx speech recognition engine had been used more than any other speech recognition algorithm and tool. In terms of the games, 48.15% had been designed in a way that children could practice with the help of their parents. The evaluation of games showed a positive effect on children's satisfaction, motivation, and attention during speech therapy exercises. The biggest barriers and challenges mentioned in the studies included sense of frustration, low self-esteem after several failures in playing games, environmental noise, contradiction between games levels and the target group's needs, and problems related to speech recognition.

**Conclusion:**

The results of this study showed that the games positively affect children's motivation to continue speech therapy, and they can also be used as the SLPs' aids. Before designing these tools, the obstacles and challenges should be considered, and also, the solutions should be suggested.

## 1. Introduction

Speech is considered as one of the important means of human communication, and, among the other ways of communication such as writing, body language, and gesture, it is the most direct and principal method of communication [1]. Speech could also be considered an acoustic signal that expresses what is in the human mind [2]. The term “speech disorder” refers to a problem in the production of speech sounds (articulation disorder), fluency, phonation (voice disorders), and resonance of speech [3, 4].

Early intervention for people who suffer from speech disorders would prevent many problems in the future which could lead to poor academic performance, reduced quality of life, reduced job opportunities, negative social consequences, impaired social interaction, and lack of independence [5–7]. Persistent communication problems can eventually lead to psychiatric disorders and anxiety in particular [8]. The high complexity of speech and language development and its relationship with the development of the other areas such as cognitive and physical developments reveal the importance of early intervention for children suffering from speech impairments [7]. In addition, some disorders, such as stuttering, if left untreated in childhood, can become a chronic disorder that causes many problems in adulthood [9]. Some other disorders may require intensive treatment over a period of time, such as hearing impairments, which have recently been treated by cochlear implant surgery [10, 11]. Therefore, providing speech therapy services for these people should be facilitated by finding new treatment presentation methods. For speech therapy exercises to be effective, therapy sessions must be individualized, frequent, and intensive [12]. In-person referral to speech and language pathologists (SLPs) is not possible due to various reasons such as the limitation in the number of therapists, geographical location, and economic conditions, especially for people living in rural and low-income areas [13]. One of the ways to solve these problems is to provide children with speech therapy exercises at home. However, home therapies would result in two challenges; firstly, children must be constantly motivated to perform exercises that are often monotonous and repetitive, and, secondly, during the practice, the feedback is presented via an adult that is not convenient for children [14]. In order to manage these challenges, digital games that have the ability to motivate and give suitable feedback to their users could be applied.

Today, digital games have been used in various areas for purposes other than entertainment [15]. Health care is one of these areas in which digital games have been used in education and training, treatment, rehabilitation, and health promotion. These games are referred to as serious games [15]. Serious games can be used anywhere and anytime and have the ability to provide an interactive environment for training different skills to users. Studies show that serious games are potentially effective tools in promoting knowledge and training various skills [16]. Because of the following features, serious games would be widely used in the field of education and treatment of children: (1) emotional attachment that is created between characters, game environment, and players, which leads to the continuation of the treatment process; (2) the positive and negative feedback that players receive in proportion to the actions they take; (3) the motivation and encouragement that children receive for their success in games; and (4) the rules that cause education or treatment to be done with certain principles [17].

In recent decades, the number of studies, which have used new technologies such as games for rehabilitation, has increased [18–20]. The field of speech therapy is not an exception, and many studies have designed games in this field to treat speech disorders [21–23]. The nature of digital and digital games is to provide feedback to users while playing. Games that develop in speech therapy are usually controlled by speech recognition to treat these disorders effectively. Due to these games being helpful, they must go beyond a simple mobile application and, for example, be able to compare the pronunciation of a word by a child with speech disorder with its correct pronunciation and give the necessary feedback to encourage the child to continue playing.

Numerous review studies have been conducted to integrate and give a unified view of the application of games in various areas of health care, among which is the scoping review of Koutsiana et al. [24] that investigated the effect of serious games technology in the upper extremity. Another one is Craig's study [25], which was conducted to examine serious games in children with autism.

There have also been several review studies in speech therapy. Furlong et al., in 2018, conducted a study to review mobile apps designed to treat speech disorders in children. This study's purpose was only to examine Google Play's and Apple iTunes Stores' applications, and studies published in this area have not been reviewed [26]. Another systematic review study was conducted by McKechnie et al. to examine the use of Automated Speech Analysis (ASA) tools to analyze and correct speech in children who are learning a foreign language and children with speech disorders [27]. None of the mentioned studies focused on published studies of digital games designed for speech therapy in children.

There are several questions and ambiguities about the games developed in the field of speech therapy for children which include the following:

1. In general, how many studies about games have been published in speech therapy for children in ISI Web of Science, PubMed, Scopus, and IEEE, and what is the purpose (no app store)?
2. What types of speech disorders in children have these games been developed for, and which speech skills have been taught?
3. What results have been obtained from the evaluation of these games developed for children, and have they met the goals set by the developers?
4. To what extent have the developed games for children been able to help SLPs to reduce their workload?
5. In what languages have the speech therapy games been developed for children, and in which countries have they been used?
6. What challenges have the speech therapy game developer for children encountered so that identifying them can guide other researchers in predicting the problems?

Therefore, the present study aims to answer the above ambiguities and questions through a systematic and comprehensive review of the games developed in this area.

## 2. Materials and Methods

### 2.1. Search Strategy

Four databases of Medline (through PubMed), Scopus, Web of Science, and IEEE Xplore were investigated to find articles on designing speech therapy games for children. Articles published by July 14, 2021, were retrieved using a combination of related MeSH terms and free-text keywords. The applied keywords consisted of three clusters (speech, children, and game), shown in Table 1. The keywords used were matched to the search strategy in each database. In the search conducted on the three databases of PubMed, Scopus, and Web of Science, no filter was applied, and, in the IEEE Xplore database, only the option of “book” was removed. No time limit was applied in the search. References to all related articles were also reviewed to find if an article had not been retrieved through the search.

### 2.2. Study Selection

In the search phase, no criteria (time, language, type of printing, etc.) were applied, but the inclusion and exclusion criteria were applied in screening the title and abstract. The following inclusion and exclusion criteria were applied to enter articles in the systematic review.

Inclusion criteria were as follows:

- Peer-reviewed studies and conference papers
- Articles that reported digital games to treat speech disorders in children
- English language articles
- Articles on the games that provide feedback for children while playing them
- Games that were based on speech

Exclusion criteria were as follows:

- Articles that focused only on language disorders (disorders that involve the processing of linguistic information) and did not target children's speech in general
- Articles designed for teens and adults
- Articles that did not focus on speech therapy and were intended to diagnose speech disorders
- Articles that were only in the form of an application and did not give any feedback to the child
- Non-English-language articles
- Review studies, letters, study protocols, books, thesis, and monographs
- Repeated articles extracted from a study
- “Low quality” articles based on the Joanna Briggs Institute (JBI) quality assessment checklist

### 2.3. Quality Assessment

The quality of retrieved articles was assessed by the JBI checklist. The JBI's critical appraisal tools can be used to evaluate the quality of a wide range of published articles, including systematic reviews, case controls, case reports, cohorts, randomized controlled trials, and qualitative studies. The purpose of this appraisal is to assess the methodological quality of studies and to determine the extent to which a study has addressed the possibility of bias in its design, implementation, and analysis.

In this study, the JBI checklist for nonquantitative studies was used. This checklist has ten questions in the following order:

1. Is there congruity between the stated theoretical perspective and the research methodology?
2. Is there congruity between the research methodology and the research question or objectives?
3. Is there congruity between the research methodology and the methods used to collect data?
4. Is there congruity between the research methodology and the representation and analysis of data?
5. Is there congruity between the research methodology and the interpretation of results?
6. Is there a statement locating the researcher culturally or theoretically?
7. Is the researcher's influence on the research, and vice versa, addressed?
8. Are participants, and their voices, adequately represented?
9. Is the research ethical according to current criteria, or for recent studies, and is there evidence of ethical approval by an appropriate body?
10. Do the conclusions drawn in the research report flow from the analysis or interpretation of the data?

These questions can be answered with four options: 1: yes, 2: no, 3: unclear, and 4: not applicable.

Each “yes” answer corresponds to one score, so the sum of the scores will be between 0 and 9 for each study (question 6 was not applicable for this study). Based on Chisini et al.'s study [28], points were totaling as follows:

  0–3: low quality
  4–6: medium quality
  7–9: high quality

### 2.4. Data Extraction

The search results from the four databases were entered into Endnote (references management software). In the first stage, the titles and abstracts of the articles were independently reviewed by three authors (SS, MSSP, and HB) under the supervision of MGS, and the articles that seemed relevant entered the full-text review. Before extracting the data from the full texts of the articles, interrater reliability check between the evaluators was performed. At this stage, 40% of the included articles and 20% of the excluded articles by two authors from three authors were randomly selected, and interrater reliability checks were performed. There was no disagreement between the reviewers. In the next step, the full texts of the related articles were retrieved, and three authors examined their quality with the JBI checklist. In the third stage, after determining the quality of the articles, each of the retrieved articles was reviewed in terms of the inclusion and exclusion criteria, and the articles that met the inclusion criteria were entered into the systematic review. Finally, an Excel form was designed, and the following information was extracted from each of the articles and entered into the relevant form:

The study, year of publication, country, target group, type of speech skill, children age ranges, type of support, supported language level (phoneme/syllable/word/phrase/sentence), type of evaluation, results of evaluation, name of the game, platform, supported language, target phonemes, 3D or 2D, game engine, training required to use the game, speech recognition technology or system, and frame of the game.

In the data extraction stage, interrater reliability was investigated between the authors. The two authors disagreed on approximately 4% of the information extracted. Disagreement between the authors was resolved by discussion with MGS.

Also, several terms have been used in this study, which are defined as follows:

  Type of support: In this study, this variable refers to whether the child can use the developed game with the help of parents or whether it must be used with the help of the therapist
  Supported language level: In this study, we considered five levels, including phoneme/syllable/word/phrase/sentence and then expressed to what level the developed games can train speech to children with speech disorders
  Supported language: This variable refers to the language of the designed game, for example, English, Portuguese, Japanese, and Persian

### 2.5. Data Analysis

Due to the qualitative data extracted in this study, no meta-analysis was performed, and the results were reported as a narrative synthesis of evidence. Excel software was used to provide descriptive statistics in the form of frequency and percentage. The games as 2D or 3D were determined based on the studies' narratives, and, in some studies, they were determined based on the images used in these studies. The age range of children who were the target group of designed games had also been explicitly mentioned in some studies, and this was not the case in some other studies. However, to give a perspective to other researchers, parents, and SLPs who intend to use the games designed in the field of speech therapy, the age range of people who participated in the evaluation of these games had been mentioned. The type of platform mentioned in some studies as laptops, windows, and computers was considered PC, and, in studies where the platform had been mentioned as tablets and smartphones, it was considered mobile. In this study, the country of the first author is mentioned.

## 3. Results

### 3.1. The Results of the Literature Search

**Figure 1:**
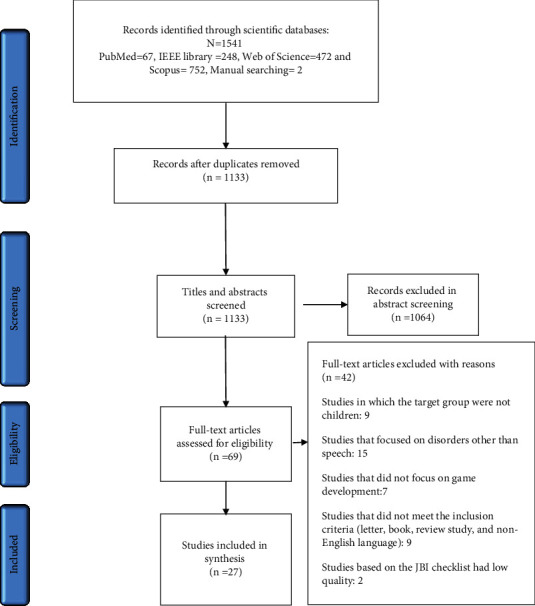
Flow diagram of the literature search and study selection.

**Figure 2:**
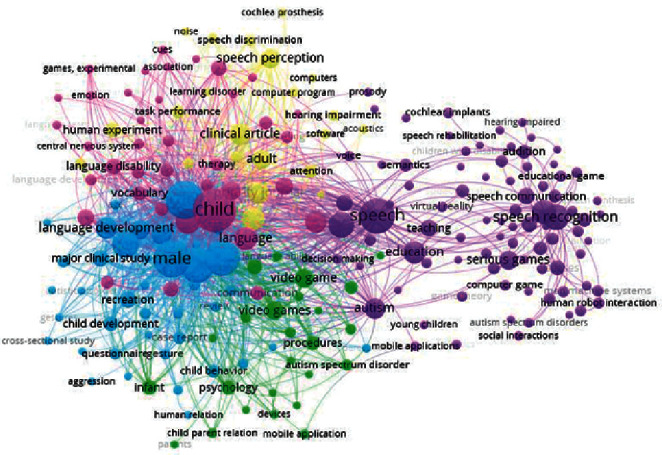
Keyword cooccurrence in VOSviewer software.

The process of searching and selecting articles based on Preferred Reporting Items for Systematic Reviews and Meta-Analyses (PRISMA) diagram is shown in Figure 1. A systematic search through four databases resulted in the retrieval of 1541 articles, of which 1133 remained after the removal of duplicates. Screening of the titles and abstracts of studies resulted in the removal of 1064 articles. The full texts of 69 articles seemed relevant. After reviewing the 69 articles, 27 that were eligible for entering the study based on the JBI checklist, inclusion criteria, and exclusion criteria were selected, and the desired information was extracted from them.  Table 2 shows the quality assessment results of articles using the JBI checklist. According to the quality assessment, only medium- and high-quality studies were included in this systematic review, of which 62.96% were of high quality, and 37.04% were of medium quality out of 27 studies. To illustrate the frequency of words in the titles and abstracts of the articles under review, the cooccurrence of keywords was determined by VOSviewer software (Figure 2).

### 3.2. General Characteristics of the Selected Studies

**Figure 3:**
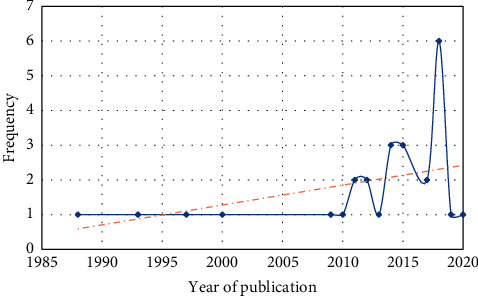
The distribution of studies based on the year of publication.

The key features of the studies included in the systematic review are summarized in Tables 3 and 4. The distribution of studies by year of publication is shown in Figure 3. The oldest study was conducted in 1988, and the most recent one was conducted in 2020. Most studies (*n* = 6) had been conducted in 2018.

**Figure 4:**
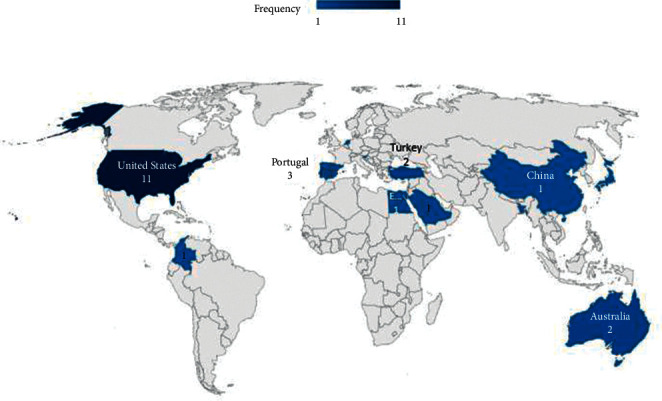
The distribution of articles based on countries.

The studies included in this review had been conducted in 13 different countries (country of the first author). The distribution of articles by country is shown in Figure 4 on the world map. As shown in the map, USA with 11 studies (40.7%) had designed the highest number of games in the field of speech therapy.

The age range of children, who used these games, had not been mentioned in nine studies (33.33%), and, in 18 studies where it had been mentioned, the games had been designed for the age range of 2 to 14 years.

### 3.3. Description of the Features of Speech Therapy Games

**Figure 5:**
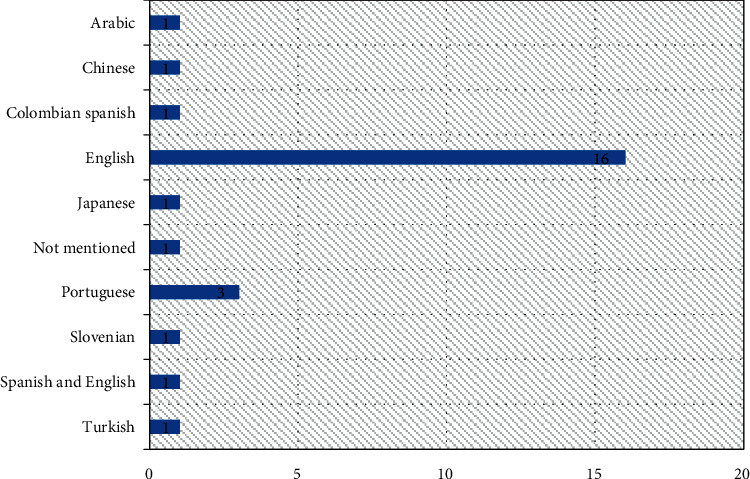
Language of designed games for speech therapy.

The results of this review showed that the games had been designed in 9 different languages, among which more than 50% were designed in English (*n* = 16, 59.25%) (Figure 5).

The results also showed that, in 19 studies (70.4%), there was no indication of which phonemes had been taught, and only eight studies mentioned the target phonemes.

The 2D and 3D of the designed games had not been mentioned in several studies and could not be determined from the images in the studies (*n* = 5, 18.5%). Most of the designed games were 2D (*n* = 19, 70.4%), and only three (11.1%) were 3D games.

In 19 studies (70.4%), the game engine used to develop speech therapy games had not been mentioned, but, in the eight studies that had mentioned it, the “Unity” engine had the highest frequency with five studies (18.5%).

The speech recognition process requires different technologies and algorithms. In 15 studies (55.55%), the type of technology used had not been mentioned. Among the technologies mentioned, the MFCC algorithm and the PocketSphinx speech recognition engine were used in three studies.

**Figure 6:**
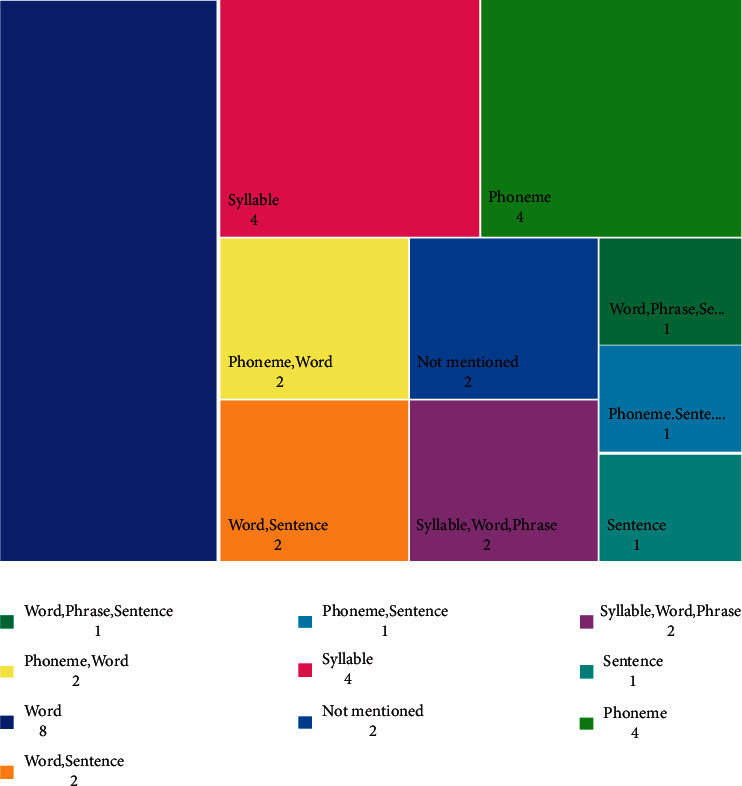
Characteristics of games based on language levels from phoneme to sentence.

The designed games trained different language levels, including five levels of phoneme, syllable, word, phrase, and sentence. None of the games trained all levels from phoneme to sentence, and only one to three levels were taught by them (Figure 6). Of the 25 studies that mentioned the level of training, eight studies (29.6%) trained words, and eight studies trained phonemes and syllables (four studies each).

### 3.4. Target Group, Skills, and Platform of the Designed Games

**Figure 7:**
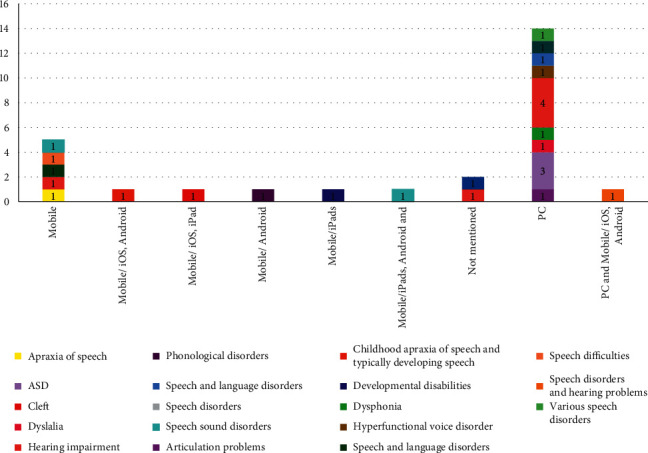
Distribution of platforms based on target population.

The target groups of the designed games were different patients, but children with hearing impairments and autism spectrum disorders attracted the most attention of researchers with six and three studies, respectively. The game platform had not been mentioned in two studies. In 14 studies, the relevant platform was PC (52%), and, in 10 studies (37.04%), speech therapy games had been developed on a mobile platform, and one study had covered both mobile and PC platforms.  Figure 7 shows the designed games based on the target group and platform, and we can see on which platform the games are available for each disease.

This study showed that speech therapy games had been used to train different speech skills, but the main focus of these games was on articulation, which with 12 studies accounted for a total of 44.5% of the studies.

### 3.5. Level of Children's Independence in Using Games

The level of support that each of the speech therapy games provided for children's independence in using speech therapy tools is presented in Table 5. It should be noted that 48.15% of speech therapy games had been designed so that children could practice speech skills with the help of parents at home.

### 3.6. Training Required to Use the Game

In 10 studies (37.04%), it was mentioned that children were trained to use the developed game, and, in other studies (17 studies), children's training was not described.

### 3.7. Challenges and Obstacles of Speech Therapy Games

The challenges and obstacles that studies have encountered in designing and using speech therapy games are listed in Table 6. Most of the challenges mentioned in the studies include sense of frustration and low self-esteem after several failures in playing games, environmental noise that leads to reduced game performance, and contradiction between game levels and the needs of target groups.

## 4. Discussion

This study examined the games designed for children with speech disorders. For this purpose, a search was conducted in four valid databases, and several related articles were found, which were later narrowed down to 69 articles based on inclusion and exclusion criteria. By reviewing the full texts of these 69 articles, 27 of them met the criteria to enter the systematic review, and the target information was extracted from them. This study showed that more than half of the games had been designed in English language, and the most common technologies used for speech recognition were PocketSphinx and the MFCC algorithm. Autistic patients and children with hearing impairments have received more attention from researchers. The designers of these games faced many challenges and limitations, such as environmental noise, problems with speech recognition, a sense of frustration in children after several failures in playing the game, and the incompatibility of the game levels and the needs of the target group. Among the games, 48.15% had been designed so children could practice with their parents during speech therapy exercises. The designed games had been evaluated in different ways, and the results showed that the games had a positive effect on children's motivation, satisfaction, and attention.

One of the challenges that researchers face in developing speech therapy games is the problem with speech recognition. This study showed that several speech recognition algorithms and engines had been used, the most common of which include PocketSphinx speech recognition tool and MFCC algorithm. PocketSphinx has many advantages for researchers, such as being free and having real-time recognition ability [53], but it also has limitations such as lack of support for speech recognition in all languages. This speech recognition tool only supports several languages, including English (American and British English), Chinese, French, Spanish, German, and Russian [54], and this problem causes researchers to use different algorithms. Classifiers and various techniques can be used to detect speech, including Hidden Markov Models (HMM), Artificial Neural Networks (ANN), Support Vector Machines (SVM), Mel-Frequency Cepstral Coefficients (MFCC), Vector Quantization (VQ), Linear Predictive Coding (LPC), Perceptual Linear Prediction (PLP), and PLP-RASTA (PLP-Relative Spectra) [2, 55]. The MFCC, PLP, and LPC are among the algorithms that are widely used in the field of speech processing [2]. The above algorithms can be used to extract features from acoustic signals. Algorithms such as Dynamic Time Warping (DTW) can also be used for feature matching and comparison between stored patterns and recorded speech [12, 55]. Therefore, the above algorithms are suggested to be used to solve problems related to speech recognition and lack of support of speech recognition tools.

Environmental and ambient noise was another challenge found in this study, which needs to be controlled by the developer of speech therapy games. If children use the developed games at home, parents may not be able to control the ambient noise, which can reduce the performance of speech recognition tools [56]. Through noise reduction, speech recognition can be improved by 15 to 25% [57], so game designers should consider this factor and control ambient noise. Numerous studies have used different methods to control environmental noise. For instance, Nasiri et al. [35] used Noise Plotter to detect ambient noise during recording. Gomez et al. [58] proposed a spectral subtraction-based method to eliminate environmental noise, and Betkowska et al. [59] used Factorial Hidden Markov Model (FHMM) to increase speech recognition accuracy in noisy environments. Thus, the effect of environmental noise on the performance of the designed tool and its control should be considered by designers.

The results of this study showed that a high percentage of the games had been designed in a way where children would be able to practice speech therapy requested by SLPs with the help of parents at home. Due to the high workload of SLPs, the lack of therapists, and the large number of sessions that these children have to practice, the creation of such tools can reduce the workload of therapists [60] and highlight the role of parents in helping their children's treatment.

One of the important issues that various studies have pointed out was the sense of frustration and low self-esteem of children after several failures in playing games, which could negatively impact users' feelings, the overall quality of the game, and its success [61]. The factors that lead to a decrease in children's self-esteem and an increase in their sense of frustration include lack of children's speech recognition after several attempts, excessive difficulty of playing for children, lack of achieving the desired level in the allotted time frame, lack of variation in games, and invariable graphical themes [14, 39, 44, 47]. Therefore, in order to create a successful game in the field of speech therapy, we need to balance speech exercises with other factors such as level of challenge, game mechanics, game difficulty, how to design a graphical interface, and how to create an attractive game for children to increase their interaction with the game [14, 44]. Games should also be designed to respond to the user's emotional state [62]. Therefore, researchers should be fully acquainted with the principles of game design and consider several points during their design to avoid such problems.

The present study has several strengths and limitations. This study includes the methods used to reduce publication bias, which include a comprehensive search in several valid databases with no time limit (i.e., Medline, ISI, Scopus, and IEEE Explore), removal of repeated articles extracted from a single study, use of articles published in conferences, and quality assessment of the articles. However, non-English-language articles were not reviewed in this study, which could be a limitation.

In future studies, the games designed in the field of language disorders are suggested to be examined, and the games designed for speech disorders in adults (e.g., speech disorders after stroke). The cost-effectiveness of games design in speech therapy could also be considered.

## 5. Conclusion

Technology interventions, including games, have the potential to be used as the SLPs' adjunct tools to treat speech disorders. This can reduce therapists' workload and direct treatment sessions to children's homes, enabling them to practice under parental supervision. In children, games, because of their appeal and entertaining nature, can lead to the continuation of the treatment process. Before designing games, the researchers should pay careful attention to the various challenges that may arise in the design of these tools, and the appropriate solutions should be considered. Familiarity with the principles of game design is essential and can be effective in the continuous use of these games.

## Figures and Tables

**Table 1:** Keywords related to game, speech, and children.

Domain	Keywords
Game	(“Video games”[mesh] OR “game” OR “games” OR “gamification” OR “video game” OR “computer games” OR computer game”)
Speech	(“Speech therapy”[mesh] OR “speech therapies” OR “therapies, speech” OR “therapy, speech” OR “speech”[mesh] OR “public speaking” OR “speaking, public” OR “speech disorders”[mesh] OR “speech disorder rehabilitation” OR “voice training”[mesh] OR “training, voice” OR “trainings, voice” OR “voice trainings” OR “cleft palate”[mesh] OR “cleft palates” OR “palate, cleft” OR “palates, cleft” OR “stuttering” OR “voice disorders”[mesh] OR “voice disturbance” OR “voice fatigue” OR “voice fatigues” OR “neurologic voice disorder” OR “neurologic voice disorders” OR “articulation disorders”[mesh] OR “phonological impairments” OR “phonological impairment” OR “phonology impairment” OR “phonology impairments” OR “disarticulation disorder” OR “disarticulation disorders” OR “misarticulation” OR “unintelligible articulation” OR “unintelligible articulations”)
Children	(“Child”[mesh] OR “children” OR “child, preschool”[mesh] OR “preschool child” OR “children, preschool” OR “preschool children”)

**Table 2:** Results of quality assessment of articles with JBI checklist.

Study	Questions										
	*Q*1	*Q*2	*Q*3	*Q*4	*Q*5	*Q*6	*Q*7	*Q*8	*Q*9	*Q*10	Final score
Takagi et al. [29]	Y	Y	Y	N	N	N/A	UC	Y	N	Y	5 (medium)
Lopes et al. [30]	Y	Y	Y	Y	Y	N/A	Y	Y	N	Y	8 (high)
Duval et al. [31]	Y	Y	Y	Y	Y	N/A	Y	Y	N	N	7 (high)
Zajc et al. [32]	Y	Y	Y	Y	N	N/A	UC	UC	Y	Y	6 (medium)
Hair et al. [21]	Y	Y	Y	Y	Y	N/A	Y	Y	Y	Y	9 (high)
Elhady et al. [33]	Y	Y	Y	Y	Y	N/A	Y	Y	N	Y	8 (high)
Anjos et al. [34]	Y	Y	Y	Y	Y	N/A	Y	Y	N	Y	8 (high)
Ahmed et al. [14]	Y	Y	Y	Y	Y	N/A	Y	Y	Y	Y	9 (high)
Nasiri et al. [35]	Y	Y	Y	Y	Y	N/A	N	UC	N	Y	6 (medium)
Madeira et al. [36]	Y	Y	Y	Y	Y	N/A	Y	Y	N	Y	8 (high)
Fardoun et al. [37]	Y	Y	Y	Y	Y	N/A	UC	UC	N	Y	6 (medium)
Cler et al. [38]	Y	Y	Y	Y	Y	N/A	Y	N	Y	Y	8 (high)
Liu et al. [39]	Y	Y	Y	Y	Y	N/A	N	N	N	Y	6 (medium)
Rubin et al. [40]	Y	Y	Y	Y	Y	N/A	Y	Y	N	Y	8 (high)
Navarro-Newball et al. [41]	Y	Y	Y	Y	Y	N/A	Y	Y	Y	Y	9 (high)
Lan et al. [42]	Y	Y	Y	Y	N	N/A	UC	N	N	N	4 (medium)
Tan et al. [43]	Y	Y	Y	Y	Y	N/A	Y	N	N	N	6 (medium)
King et al. [44]	Y	Y	Y	Y	Y	N/A	Y	N	Y	Y	8 (high)
Cagatay et al. [45]	Y	Y	Y	Y	Y	N/A	Y	N	N	Y	7 (high)
Rahman et al. [46]	Y	Y	Y	Y	Y	N/A	UC	N	N	Y	6 (medium)
Frutos et al. [23]	Y	Y	Y	Y	Y	N/A	Y	N	N	Y	7 (high)
Umanski et al. [47]	Y	Y	Y	Y	Y	N/A	Y	N	N	Y	7 (high)
Hoque et al. [48]	Y	Y	Y	Y	Y	N/A	Y	Y	N	Y	8 (high)
Bunnell et al. [49]	Y	Y	Y	Y	Y	N/A	Y	N	N	N	6 (medium)
Soleymani et al. [50]	Y	Y	Y	Y	Y	N/A	Y	N	N	Y	7 (high)
Javkin et al. [51]	Y	Y	Y	Y	Y	N/A	UC	N	N	N	5 (medium)
Mahshie et al. [52]	Y	Y	Y	Y	Y	N/A	UC	Y	N	Y	7 (high)

Y: yes; N: no; UC: unclear; N/A: not applicable.

**Table 3:** The characteristics of reviewed articles.

Authors, year	Country	Target group	Type of speech skill	Children age ranges	Type of support	Supported language level (phoneme/syllable/word/phrase/sentence)	Type of evaluation	Results of evaluation
Takagi et al. [29]	Japan	Children with hearing impairment	(i) Vocalization	Pre-school children	Children can practice with parents at home	(i) Word	Not evaluated	Not evaluated
Lopes et al. [30]	Portugal	Children with dysphonia	(i) Sustained vowel exercise	4-5 years old	Therapist involved	(i) Phoneme	Test with users	The game gives children the motivation to continuing practicing.
Duval et al. [31]	USA	Children with developmental disabilities	(i) Articulation	Not mentioned	Not mentioned	(i) Word	Usability evaluation	This study yielded refined functional requirements based on user feedback, relevant reward systems to implement based on user interest, and insights on the preferred hybrid game structure.
						(ii) Phrase		
						(iii) Sentence		
Zajc et al. [32]	Slovenia	Children with speech and language disorders	(i) Phonological awareness exercise	3–12 years old	Not mentioned	(i) Phoneme	Test with users	The game had positive impact on the children's motivation and satisfaction.
Hair et al. [21]	USA	Children with speech sound disorders	(i) Articulation	4–12 years old	Children can practice with parents at home	(i) Word	Test with users	The results indicate that game successfully engages children and speech exercises. Children are willing to complete the required speech exercises while playing a game they enjoy.
Elhady et al. [33]	Egypt	Children with dyslalia	(i) Articulation	7–10 years old	Children can practice with parents at home	(i) Word	Test with users/evaluation performance of the speech recognition system	A noticeable progress in children dyslalia appeared with the proposed system/recognition accuracy: 82.1–95.6.
Anjos et al. [34]	Portugal	Children with speech sound disorders	(i) Articulation	5–9 years old	Children can practice with parents at home	(i) Phoneme	Giving feedback from children and SLP	The feedback from children confirmed that children liked the game. The SLPs showed interest in game and considered it a good method for children training.
Ahmed et al. [14]	Australia	Children with childhood apraxia of speech and typically developing speech	(i) Articulation	6–11 years old	Children can practice with parents at home	(i) Word	Feasibility study/evaluation performance of the speech recognition system	Children and SLPs found speech-controlled games interesting and fun/ASR accuracy: specificity: 77%; sensitivity: 51%.
Nasiri et al. [35]	Turkey	Children with speech disorders and hearing problems	(i) Articulation	2–6 years old	Children can practice with parents at home	(i) Word	Not evaluated	Not evaluated
Madeira et al. [36]	Portugal	Children with phonological disorders	(i) Metaphon therapy	3–8 years old	Not mentioned	(i) Phoneme	Usability evaluation	Super-Fon's usability was acceptable and very near to a good ranking.
						(ii) Word		
Fardoun et al. [37]	Saudi Arabia	Children with speech difficulties	(i) Respiratory exercises	Not mentioned	Children can practice with parents at home	(i) Phoneme (ii) Word	Not evaluated	Not evaluated
			(ii) Labial exercises					
			(iii) Vocalization exercises					
Cler et al. [38]	USA	Children with velopharyngeal dysfunction	(i) Nasalization practice	4–14 years old	Children can practice with parents at home	(i) Word	Pilot testing with users	Over 90% of the participants reported that the game was at least “kind of fun” and the equipment was at least “kind of comfortable.”
Liu et al. [39]	China	Children with hearing impairment	(i) Articulation	Not mentioned	Children can practice with parents at home	(i) Word	Usability evaluation	They had an emotional value recovery.
						(ii) Sentence		
Rubin et al. [40]	USA	Children with cleft	(i) Not mentioned	2–5 years old	Children can practice with parents at home	(i) Phoneme	Pilot testing with users	Children enjoyed the game but grew bored due to the delays of phrase-based speech recognition.
						(ii) Sentence		
Navarro-Newball et al. [41]	Colombia	Children with hearing impairment	(i) Articulation	3–11 years old	Not mentioned	(i) Syllable	Informal summative evaluation for assessing user experience/evaluation performance of the speech recognition system	The results of evaluation showed it to be a suitable tool to maintain the attention and enthusiasm in repetitive tasks. Correct percentage: 80.51
Lan et al. [42]	USA	Children with apraxia of speech	(i) Timing and vocal loudness exercise	4–12 years old	Not mentioned	(i) Not mentioned	Pilot testing with users	Results support the feasibility of the game as a speech training tool.
Tan et al. [43]	Australia	Children with speech disorders	(i) Vocalization	Not mentioned	Not mentioned	(i) Word	Pilot testing with users	The children appear to be engaged and interested in playing.
King et al. [44]	USA	Children with hyperfunctional voice disorder	(i) Resonance voice exercise	School-age children	Not mentioned	(i) Syllable	Feasibility study	This study found that a purely entertaining video game can be implemented as a voice therapeutic protocol.
						(ii) Word		
						(iii) Phrase		
Cagatay et al. [45]	Turkey	Children with speech and language disorders	(i) Not mentioned	Not mentioned	Therapist involved	Not mentioned	Pilot testing with users	They showed increasing interest in the game.
Rahman et al. [46]	Bangladesh	Children with ASD	(i) Making intelligible sounds and correct sentences with words	Not mentioned	Therapist involved	(i) Word	Pilot testing with users	The results indicated the supremacy of gaming method for learning worlds quickly and efficiently.
						(ii) Sentence		
Frutos et al. [23]	Spain	Children with ASD	(i) Articulation	Not mentioned	Not mentioned	(i) Word	Evaluation performance of the speech recognition system	The game had good accuracy (100%).
Umanski et al. [47]	Netherlands	Children with various speech disorders	(i) Speech rhythm exercise	4–6 years old	Therapist involved	(i) Syllable	Usability evaluation	The result showed that although the game prototype requires improvement, the initiative is very welcome, and further prototypes will be anticipated.
Hoque et al. [48]	USA	Children with ASD	(i) Improving speech intelligibility	Not mentioned	Therapist involved	(i) Sentence	Pilot testing with users	Preliminary results demonstrated that the game is engaging and effective.
Bunnell et al. [49]	USA	Children with articulation problems	(i) Articulation	4–7 years old	Therapist involved	(i) Syllable	Evaluation performance of the speech recognition system	The difference in log likelihood between /r/ and /w/ models correlates well with perceptual ratings of utterances containing substitution errors but very poorly for correctly articulated examples.
						(ii) Word		
						(iii) Phrase		
Soleymani et al. [50]	USA	Children with hearing impairment	(i) Articulation	Young school-aged	Children can practice with parents at home	(i) Syllable	Test with users	The system's operation was found to be reliable.
Javkin et al. [51]	USA	Children with hearing impairment	(i) Articulation	Not mentioned	Children can practice with parents at home	(i) Phoneme	Pilot testing with users	The game was proving highly motivating to the children and encouraged them to experiment with their speech production.
Mahshie et al. [52]	USA	Children with hearing impairment	(i) Sustained vocalization	3–11 years old	Children can practice with parents at home	(i) Syllable	Test with users	The game was found to be easily incorporated into clinic activities and useful for diagnosis and therapy.
			(ii) Production of repeated syllables					
			(iii) Control of voice intensity and fundamental frequency					

**Table 4:** The characteristics of the applied games in the reviewed articles.

Study	Name of game	Platform	Supported language	Target phonemes	3D/2D	Game engine	Speech recognition technology or system	Training required to use the game	Frame of the game
Takagi et al. [29]	Not mentioned	(i) Mobile	Japanese	Not mentioned	2D	Unity	(i) Julius	Not mentioned	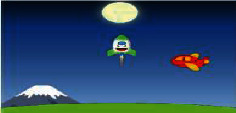
Lopes et al. [30]	Not mentioned	(i) PC	Portuguese	/A/, /e/, /i/, /o/, /u/	Not mentioned	Not mentioned	(i) MFCC	The children were trained.	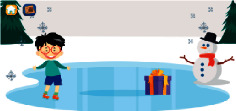
Duval et al. [31]	SpokeIt	(i) Mobile/iPads	English	Not mentioned	2D	Not mentioned	(i) PocketSphinx	The children were trained.	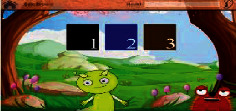
Zajc et al. [32]	Not mentioned	(i) Mobile	Slovenian	/s/, /z/, /ts/, /sh/, /ʒ/, /ch/	2D	Not mentioned	(i) Not mentioned	Not mentioned	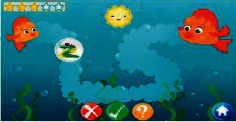
Hair et al. [21]	Apraxia world	(i) Mobile	English	Not mentioned	2D	Unity	(i) Wizard of Oz	The children were trained.	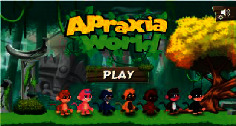
Elhady et al. [33]	Kalemni Aktar	(i) PC	Arabic	/s/, /r/	Not mentioned	Not mentioned	(i) A baseline acoustic model was trained using the Egyptian corpus.	Not mentioned	Not mentioned
Anjos et al. [34]	Not mentioned	(i) Mobile/iPads, Android and iOS	Portuguese	/s/, /z/, /sh/, /zh/	2D	Unity	(i) MFCC	The children were trained.	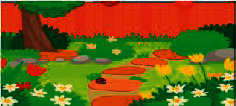
Ahmed et al. [14]	Not mentioned	(i) Mobile/iOS, Android	English	Not mentioned	2D	Not mentioned	(i) PocketSphinx (American English)	The children were trained.	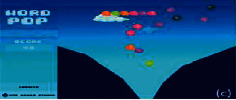
Nasiri et al. [35]	Into the Forest	(i) PC	English	Not mentioned	3D	Unity	(i) Windows UDP Voice Recognition server	Not mentioned	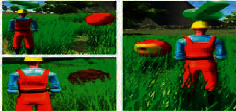
		(ii) Mobile/iOS, Android							
Madeira et al. [36]	Super-Fon	(i) Mobile/Android	Portuguese	Not mentioned	Not mentioned	Not mentioned	(i) Not mentioned	Not mentioned	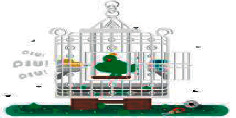
Fardoun et al. [37]	Not mentioned	(i) Mobile	English	Not mentioned	2D	Not mentioned	(i) Not mentioned	Not mentioned	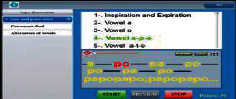
Cler et al. [38]	Not mentioned	(i) Not mentioned	English	Not mentioned	2D	Developed in C# using the .NET 4.5.1	(i) Not mentioned	Not mentioned	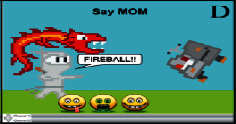
Liu et al. [39]	NewVoice	(i) Not mentioned	Chinese	Not mentioned	2D	Not mentioned	(i) Not mentioned	Not mentioned	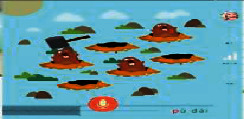
Rubin et al. [40]	Speech Adventure	(i) Mobile/iOS, iPad	English	Not mentioned	2D	Cocos2D	(i) OpenEars	Not mentioned	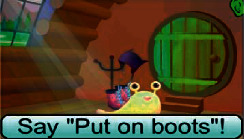
Navarro-Newball et al. [41]	Talking to Teo	(i) PC	Colombian Spanish	/d/, /t/, /n/, /s/, /l/	2D	Not mentioned	(i) Perceptual Linear Prediction (PLP) coefficients	Not mentioned	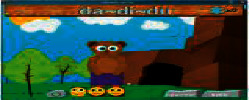
Lan et al. [42]	Flappy Voice	(i) Mobile	English	Not mentioned	2D	Not mentioned	(i) Not mentioned	Not mentioned	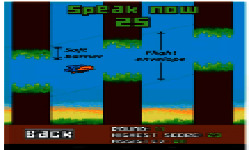
Tan et al. [43]	sPeAK-MAN	(i) PC	English	Not mentioned	2D	Microsoft's XNA Game Studio 4.0 framework	(i) Microsoft Speech SDK	Not mentioned	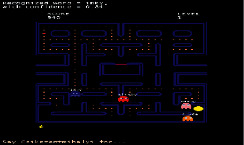
King et al. [44]	Opera Slinger	(i) PC	English	Plosive or nasal consonant	3D	Not mentioned	(i) Not mentioned	The children were trained.	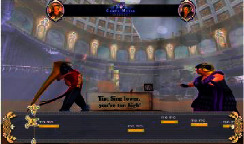
Cagatay et al. [45]	Not mentioned	(i) PC	Turkish	Not mentioned	3D	Unity	(i) Not mentioned	The children were trained.	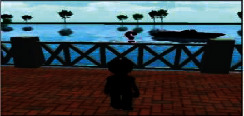
Rahman et al. [46]	Not mentioned	(i) PC	English	Not mentioned	2D	Not mentioned	(i) Microsoft Speech Engine for English Speech	The children were trained.	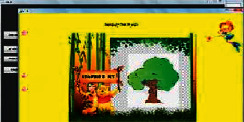
							(ii) SDK 5.1, Microsoft .NET Framework 3.5.		
Frutos et al. [23]	Not mentioned	(i) PC	Spanish and English	Not mentioned	2D	Not mentioned	(i) DTW	The children were trained.	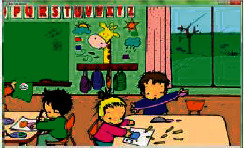
							(ii) MFCCs		
							(iii) SPHINX4		
Umanski et al. [47]	Not mentioned	(i) PC	English	Not mentioned	2D	Not mentioned	(i) Not mentioned	Not mentioned	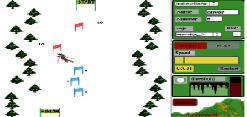
Hoque et al. [48]	Not mentioned	(i) PC	English	Not mentioned	Not mentioned	Not mentioned	(i) Sona-Speech's Multidimensional Voice Program (MDVP)	Not mentioned	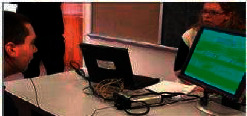
							(ii) Praat speech processing software		
Bunnell et al. [49]	Not mentioned	(i) PC	Not mentioned	/r/	Not mentioned	Not mentioned	(i) Discrete Hidden Markov Model	The children were trained.	Not mentioned
Soleymani et al. [50]	SIM	(i) PC	English	/a/, /i/, /u/	2D	Not mentioned	(i) Not mentioned	Not mentioned	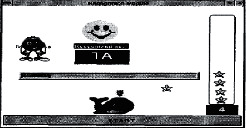
Javkin et al. [51]	Not mentioned	(i) PC	English	Not mentioned	2D	Not mentioned	(i) Not mentioned	Not mentioned	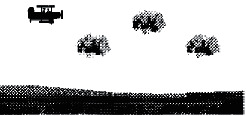
Mahshie et al. [52]	STS, SPS	(i) PC	English	Not mentioned	2D	Not mentioned	(i) Not mentioned	Not mentioned	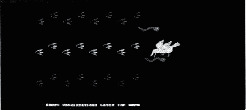

**Table 5:** The level of support provided by the games for children with speech disorders.

Type of support	Frequency	Percentage
Children can practice with parents at home	13	48.15
Not mentioned	8	29.63
Therapist involved	6	22.22
Total	27	100

**Table 6:** Challenges and obstacles of speech therapy games.

#	Challenges and obstacles	Studies
1	Sense of frustration and low self-esteem of children due to the lack of voice recognition or progress in playing game	[14, 39, 44, 47]
2	Ambient and environmental noise that affected the game performance	[30, 32, 33, 49]
3	Contradiction between game levels and the needs of target groups (the game was very difficult or too easy)	[14, 21, 52]
4	The game was challenging because it required two hands to play	[14, 21]
5	Children could not easily read words or phrases due to inadequate instruction	[14, 31]
6	Not all participants were willing to wear the headset microphone	[14, 42]
7	Delays in speech recognition	[40, 43]
8	The game did not recognize low tune voices, and children had to speak loudly	[30, 31]
9	The designed game did not provide feedback on accepting or rejecting children's voices	[31]
10	One of the challenges at design phase was that each target phrase or word had to be carefully crafted to fit into the narrative of the game and this was very time-consuming, which could result in minimal content	[31]
11	Negative beliefs of SLP due to unavailability of games for their professional needs	[32]
12	Internet connection restrictions in client-server architectures	[34]
13	The games had no “levels”	[14]
14	The children found the record buttons difficult to manage and required multiple screen taps	[14]
15	Immobility of the system due to Kinect dependence	[43]
16	Different accents led to the lack of voice recognition	[43]
17	The proposed games were heavily dependent on the instructors	[46]
18	Problem in syllable detection in real time	[47]
19	Disagreement between clinician and the game in terms of the correct pronunciation of sounds	[52]
20	Young children could not work with the designed games	[29]

## Data Availability

All data generated or analyzed during this study are included within this article.
